# Advances in Insulin Resistance—Molecular Mechanisms, Therapeutic Targets, and Future Directions

**DOI:** 10.3390/ijms26062574

**Published:** 2025-03-13

**Authors:** Byungyong Ahn

**Affiliations:** 1 Department of Food Science and Nutrition, University of Ulsan, Ulsan 44610, Republic of Korea; byahn@ulsan.ac.kr; Tel.: +82-52-259-2373; Fax: +82-52-259-1698; 2 Basic-Clinical Convergence Research Institute, University of Ulsan, Ulsan 44610, Republic of Korea

The development of insulin resistance (IR) is characterized by a series of metabolic disturbances, including, but not limited to, impaired glucose uptake, increased blood sugar levels, and disrupted lipid metabolism. These disturbances are initiated by a failure of the body’s cells, particularly those located in the liver, skeletal muscle, and adipose tissue, to respond appropriately to insulin [1]. The advent of insulin resistance is commonly associated with various modifiable lifestyle elements (e.g., suboptimal nutrition, inactivity), along with genetic predispositions, contributing to an accumulation of visceral adipose tissue and persistent low-grade systemic inflammation. This amalgamation of factors engenders an environment that impedes the normal functionality of the insulin signaling pathways [2,3]. Consequently, this impeding of insulin signaling results in a detrimental cycle, whereby the aforementioned factors contribute to a state of exacerbated metabolic dysfunction, which in turn fosters the onset and progression of conditions such as type 2 diabetes (T2D) and cardiovascular diseases (CVD). Furthermore, recent findings have indicated a potential role for insulin resistance in the pathophysiology of neurodegenerative diseases, such as Parkinson’s disease, where impaired insulin signaling has been associated with the onset and progression of the disease [4,5,6]. In recent years, an increasing body of research has been dedicated to understanding the molecular mechanisms that underlie insulin resistance, with significant advancements in our comprehension of the intricate signaling pathways involved. These signaling pathways, which are implicated in the development of this condition, include the insulin/IGF-1 axis, the PI3K/AKT pathway, and inflammation-driven processes. These pathways have been found to intricately interact with each other and with metabolic dysfunction, altered gene expression, and immune responses, collectively impairing cellular responses to insulin. As research progresses, novel therapeutic strategies are emerging, with a focus on targeting specific molecular pathways to restore insulin sensitivity, reduce inflammation, and improve metabolic outcomes [6,7,8].

The present editorial has been written with the intention of providing a more profound analysis of the recent advances concerning the comprehension of insulin resistance, particularly its function in the genesis of diverse chronic diseases. Through the investigation of the molecular mechanisms that propel insulin resistance, this endeavor seeks to elucidate novel therapeutic avenues and potential interventions. The identification of these mechanisms has the potential to facilitate the development of more efficacious treatments to combat this escalating global health concern. The insights derived from this study could result in a more encompassing strategy for the management of insulin resistance, thereby offering a glimmer of hope for the alleviation of the encumbrance that related diseases impose on global public health systems.

The Role of Insulin Resistance in Metabolic Diseases: Insulin resistance is frequently linked to a pro-inflammatory state, which is typified by elevated levels of inflammatory cytokines, such as tumor necrosis factor-alpha (TNF-α) and interleukins (ILs), in the circulating blood. These inflammatory mediators compromise the functionality of insulin receptors, consequently disrupting the signaling pathways that regulate glucose uptake by cells. The interplay between inflammation and insulin resistance contributes to the development of various diseases, including type 2 diabetes and cardiovascular disease, underscoring the necessity for effective interventions that target both facets of metabolic dysfunction. Gadd et al. (2024) highlighted the substantial impact of insulin resistance on long-term functional outcomes following stroke, underscoring its pivotal role in compromised recovery. The researchers observed that elevated serum levels of IGFBP-1, a protein frequently elevated in cases of insulin resistance, were associated with diminished outcomes, indicating that insulin resistance intensifies the deleterious consequences of stroke and impedes the recovery process. This underscores the critical need to address insulin resistance as a pivotal factor in enhancing patient rehabilitation outcomes [9]. Furthermore, the contribution of insulin resistance to neurodegenerative conditions, including Parkinson’s disease, highlights its pervasive influence across a broad spectrum of chronic diseases [10].

Ketosis and Metabolic Health: Recent research on metabolic diseases has increasingly focused on the relationship between ketosis and insulin resistance. Ketosis is a metabolic state in which the body shifts from using carbohydrates as its primary energy source to relying on fat oxidation. In a study by Cooper et al. (2023), the impact of ketosis suppression in subjects who had maintained a long-term ketogenic diet was explored. The study’s findings suggest that the suppression of ketosis can lead to adverse changes in biomarkers that are strongly associated with chronic diseases and the ageing process. Conversely, the maintenance of long-term nutritional ketosis has been shown to provide significant health benefits without any observed adverse effects. The data further suggest that prolonged suppression of ketosis may contribute to increased insulin resistance and hyperinsulinemia, as indicated by a decline in insulin sensitivity. In contrast, participants who sustained ketosis exhibited maintained insulin sensitivity. In view of these findings, it is important to note that reducing ketosis is not helpful in optimizing metabolic health, as prolonged suppression of ketosis may worsen insulin resistance. On the contrary, maintaining ketosis appears to be the most beneficial approach, as evidenced by a variety of health biomarkers. Furthermore, the sustained nutritional ketosis approach may help alleviate hyperinsulinaemia without compromising metabolic flexibility or carbohydrate tolerance in metabolically healthy individuals. The results of this study underscore the potential of nutritional ketosis as a sustainable and effective approach to promote long-term health and metabolic stability [11,12].

Molecular Mechanisms and Signaling Pathways Involved in Insulin Resistance: The molecular mechanisms underlying insulin resistance are complex and involve various signaling pathways, including those pertaining to the insulin/IGF-1 axis, PI3K/AKT pathway, and MAPK pathway. Disruptions in these pathways can give rise to impaired glucose and lipid metabolism, mitochondrial dysfunction, and compromised cellular balance. Recent studies have focused intensely on the role of insulin-like growth factor-binding proteins (IGFBPs) in regulating insulin signaling and their potential as therapeutic targets for insulin resistance. In their 2024 study, Gadd et al. examined the impact of elevated levels of IGFBP-1 on post-stroke recovery. They discovered an association between elevated IGFBP-1 levels and suboptimal recovery outcomes, suggesting that these proteins could serve as biomarkers for insulin resistance and its deleterious effects on post-stroke rehabilitation [9]. This research contributes to the growing body of knowledge about the role of IGFBPs in metabolic disorders and their potential as therapeutic targets. In a similar vein, Yang et al. (2023) investigated the function of myo-inositol (MI) in counteracting the effects of aging and insulin resistance by modulating the PI3K/AKT signaling pathway. Their findings underscored MI’s capacity to mitigate the deleterious consequences of insulin resistance, positioning it as a promising therapeutic agent for metabolic diseases. By modulating insulin signaling pathways, MI offers a novel approach to mitigating the impact of aging and insulin resistance, thereby contributing to enhanced metabolic health [13]. Ahn’s (2023) research on MondoA and ChREBP, two pivotal transcription factors in nutrient sensing and metabolism, has further expanded the existing body of knowledge on the subject of insulin resistance. These factors play a central role in regulating cellular responses to nutrient availability and insulin signaling, thereby offering crucial insights into the body’s adaptation to changes in nutrient status. By influencing glucose and lipid metabolism, MondoA and ChREBP have a significant impact on the development of metabolic diseases. Consequently, MondoA and ChREBP emerge as significant targets for therapeutic interventions aimed at addressing insulin resistance and its associated metabolic complications [14].

Sex-Specific Differences in Insulin Sensitivity and Resistance: Recent research has increasingly emphasized the importance of sex-specific variations in insulin sensitivity. Beaudry et al. (2023) developed models customized to predict insulin secretion and sensitivity based on gender, emphasizing that men and women may exhibit different responses to interventions targeting insulin resistance. These customized models hold great promise in improving treatment plans designed for individuals with metabolic conditions, thereby ensuring that therapeutic approaches are tailored to the distinctive needs of each sex [15]. The incorporation of sex-specific models is imperative for elucidating the mechanisms by which insulin resistance evolves and manifests disparately in men and women, particularly in contexts such as type 2 diabetes and cardiovascular diseases, where pronounced gender-based discrepancies in disease prevalence and progression have been documented. The acknowledgement and remediation of these disparities have the potential to result in the development of more efficacious, customized treatments, thereby enhancing outcomes for both male and female patients.

Glucagon and Its Role in Insulin Resistance: In addition to insulin, glucagon plays a pivotal role in the regulation of glucose metabolism. Neumann et al. (2023) investigated the impact of glucagon receptors on the mammalian heart, revealing a potential modification of glucagon’s influence on the cardiovascular system by insulin resistance. Their findings suggest that glucagon can affect various physiological processes, including heart rate, contraction strength, and glucose regulation. This interaction potentially contributes to the exacerbation of metabolic dysfunction in individuals suffering from cardiovascular diseases and stroke [16]. A comprehensive understanding of the interplay between glucagon and insulin in regulating metabolic pathways is imperative for the development of effective strategies to enhance insulin sensitivity. This knowledge is particularly crucial in the management of chronic conditions associated with insulin resistance, such as cardiovascular diseases and stroke.

Macrophage Populations in Skeletal Muscle and Their Impact on Insulin Sensitivity: Skeletal muscle, the body’s largest tissue that responds to insulin, plays a crucial role in insulin sensitivity. Research has demonstrated that the infiltration of macrophages, a type of white blood cell, into skeletal muscle contributes to insulin resistance. Lee et al. (2023) examined how distinct populations of macrophages within skeletal muscle affect insulin sensitivity. Their findings underscore the potential significance of macrophage polarization, with M1 macrophages promoting inflammation and insulin resistance, and M2 macrophages exhibiting anti-inflammatory effects. This dynamic may serve as a crucial regulatory factor in insulin sensitivity within skeletal muscle. A more profound understanding of the role of macrophages could potentially provide novel insights into therapeutic strategies aimed at modulating immune responses to enhance insulin sensitivity and address metabolic disorders, such as type 2 diabetes [17].

Insulin resistance is a hallmark of the pathogenesis of numerous chronic diseases, including type 2 diabetes, cardiovascular disease, neurodegenerative diseases such as Parkinson’s, and even stroke. The pervasiveness of insulin resistance and its association with a multitude of prevalent health conditions underscores its significance as a critical focus of medical research and clinical intervention. Insulin resistance is considered the underlying cause of metabolic dysregulation, which impedes the body’s capacity to utilize insulin efficiently. This impairment affects glucose and lipid metabolism, leading to a series of health complications.

An in-depth comprehension of the molecular mechanisms underlying insulin resistance is imperative to develop more effective therapeutic interventions. Recent research has underscored the significant roles played by inflammatory pathways, insulin-like growth factor-binding proteins (IGFBP-1), and nutrient-sensing transcription factors like MondoA and ChREBP in regulating insulin sensitivity and maintaining metabolic balance. The strategic targeting of these pathways holds significant therapeutic promise in the fight against insulin resistance and its extensive health implications. The recognition of sex-related variations in insulin sensitivity introduces a degree of complexity to the comprehension of insulin resistance. The establishment of models that differentiate by sex to predict insulin secretion and sensitivity, in conjunction with customized interventions, has the potential to enhance treatment outcomes by accounting for the physiological differences between males and females. These personalized approaches to managing insulin resistance demonstrate considerable promise for the development of more precise and effective therapeutic strategies. Moreover, research on dietary interventions such as ketosis underscores the significance of metabolic states in regulating insulin sensitivity. Nevertheless, the long-term ramifications of such interventions, notably for subjects presenting metabolic dysfunction, necessitate judicious evaluation and customized therapeutic regimens. A significant area of research interest pertains to the regulatory functions of macrophage populations in modulating insulin sensitivity within skeletal muscle tissue. The diverse spectrum of macrophage forms plays a crucial role in the orchestration of inflammatory responses and the regulation of insulin resistance. Classically activated, or M1, macrophages are pro-inflammatory, contributing to the exacerbation of insulin resistance. In contrast, alternatively activated, or M2, macrophages exhibit anti-inflammatory properties, suggesting the potential for therapeutic interventions that target these dynamic macrophage populations. A comprehensive understanding of these macrophage interactions within the skeletal muscle milieu is paramount for the development of therapeutic strategies that address not only metabolic dysfunction but also immune system dysfunction.

As research progresses, elucidating the complex network of metabolic pathways implicated in insulin resistance, new molecular targets for intervention are being identified. This development holds great promise for the development of innovative therapeutic strategies that aim to reverse insulin resistance, improve metabolic health, and enhance the quality of life for millions worldwide. Further exploration of these mechanisms is anticipated to yield novel treatment options for metabolic diseases, thus offering hope for ameliorated outcomes for affected populations.
