# Correction: Ahn, B. Advances in Insulin Resistance—Molecular Mechanisms, Therapeutic Targets, and Future Directions. *Int. J. Mol. Sci.* 2025, *26*, 2574

**DOI:** 10.3390/ijms26094171

**Published:** 2025-04-28

**Authors:** Byungyong Ahn

**Affiliations:** 1 Department of Food Science and Nutrition, University of Ulsan, Ulsan 44610, Republic of Korea; byahn@ulsan.ac.kr; Tel.: +82-52-259-2373; Fax: +82-52-259-1698; 2 Basic-Clinical Convergence Research Institute, University of Ulsan, Ulsan 44610, Republic of Korea

In the original publication [1], there was an error in the fourth paragraph for the Ketosis and Metabolic Health Section, which is a misrepresentation of the findings by Cooper et al. A correction has been made to the fourth paragraph, with the following alterations made to the text. Reference [12] was not cited; this citation has now been inserted in the fourth paragraph and should read as follows:

Ketosis and Metabolic Health: Recent research on metabolic diseases has increasingly focused on the relationship between ketosis and insulin resistance. Ketosis is a metabolic state in which the body shifts from using carbohydrates as its primary energy source to relying on fat oxidation. In a study by Cooper et al. (2023), the impact of ketosis suppression in subjects who had maintained a long-term ketogenic diet was explored. The study’s findings suggest that the suppression of ketosis can lead to adverse changes in biomarkers that are strongly associated with chronic diseases and the ageing process. Conversely, the maintenance of long-term nutritional ketosis has been shown to provide significant health benefits without any observed adverse effects. The data further suggest that prolonged suppression of ketosis may contribute to increased insulin resistance and hyperinsulinemia, as indicated by a decline in insulin sensitivity. In contrast, participants who sustained ketosis exhibited maintained insulin sensitivity. In view of these findings, it is important to note that reducing ketosis is not helpful in optimizing metabolic health, as prolonged suppression of ketosis may worsen insulin resistance. On the contrary, maintaining ketosis appears to be the most beneficial approach, as evidenced by a variety of health biomarkers. Furthermore, the sustained nutritional ketosis approach may help alleviate hyperinsulinaemia without compromising metabolic flexibility or carbohydrate tolerance in metabolically healthy individuals. The results of this study underscore the potential of nutritional ketosis as a sustainable and effective approach to promote long-term health and metabolic stability [11,12].

12. Cooper, I.D.; Kyriakidou, Y.; Petagine, L.; Edwards, K.; Soto-Mota, A.; Brookler, K.; Elliott, B.T. Ketosis Suppression and Ageing (KetoSAge) Part 2: The Effect of Suppressing Ketosis on Biomarkers Associated with Ageing, HOMA-IR, Leptin, Osteocalcin, and GLP-1, in Healthy Females. *Biomedicines* **2024**, *12*, 1553.

With this correction, the order of some references has been adjusted accordingly. The authors state that the scientific conclusions are unaffected. This correction was approved by the Academic Editor. The original publication has also been updated.
